# Co-producing a recovery college course: insights into a three-phase participatory process and contributions

**DOI:** 10.3389/fpsyt.2026.1893785

**Published:** 2026-09-14

**Authors:** Joanie Theriault, Anick Sauvageau, Catherine Briand, Johana Monthuy-Blanc

**Affiliations:** 1 Centre de recherche en santé durable VITAM, Trois-Rivières, QC, Canada; 2 Centre de recherche de l'institut universitaire de santé mentale de Montréal (CR-IUSMM), Trois-Rivières, QC, Canada

**Keywords:** co-production and co-learning, eating attitudes and behaviors, participatory design, program theory model, recovery college

## Abstract

**Introduction:**

This article provides a concrete example of a co-production process used to develop a Recovery College course about eating attitudes and behaviors. It has a dual purpose: (1) to document a three-phase participatory research approach underpinning course co-production and (2) to examine the contributions of this process, highlighting benefits for both the course and the experiential trainers involved.

**Methods:**

The study was conducted at Quebec’s first Recovery College using participatory design into three sequential phases: (1) a co-production phase combining collaborative and independent working sessions supported by participatory design tools (course co-production template and program theory model); (2) a co-validation phase involving a mixed-expertise consultation group; and (3) an initial experimentation phase across four learner groups over one year. A diverse qualitative corpus including audio recordings, research journal, audit trail grid of course improvements, transcripts of individual interviews with trainers and experimentation diary was analyzed using an analytical inquiry strategy.

**Results:**

The findings show that translating complementarity between experiential, clinical, and theoretical knowledge into concrete processes requires specific practices, notably open discussions, an organized work structure and clarified roles, alongside the use of complementary co-production tools. The articulation of co-production and co-validation phases strengthened capacities at two levels. At the intervention level, these processes supported the refinement, coherence, and safety of course content for a sensitive topic. At the trainers’ level, participation across phases contributed to power-sharing between trainers that contributed to the recognition and development of experiential expertise.

**Discussion:**

Detailed empirical accounts of how co-production is operationalized in Recovery College course development remain scarce. This study offers a transferable, step-by-step illustration of how complementarity work can be enacted through structured practices and tools. The proposed three-phase process and associated tools provide actionable guidance for Recovery Colleges and other health organizations seeking to implement co-production, support experiential experts, and enhance course quality when addressing sensitive topics. (277/250 mots).

## 1 Introduction

Co-production has been subject to various definitions (1). The early definition by Ostrom (2) presents co-production as a process involving various individuals from different organizations in a collaborative work towards the creation and the delivery of services. In early 2010, the New Economics Foundation positioned co-production as a partnership where professionals and citizens collaboratively share power to plan and deliver public services, acknowledging that both parties have essential contributions (3, 4). The shared power, together with the essential and equal contributions of all parties, deepened the definition of co-production. Recent studies report that co-production processes involve i) working in partnership bringing together different knowledge and expertise that may or not be complementary; ii) a shared decision-making approach, and iii) a common understanding of the objective and approach to be used to develop the initiative (5, 6).

For many health initiatives, a core component of co-production is the mobilization of experiential knowledge. In the context of Recovery College (RC) and health research, experiential knowledge refers to what individuals have learned and developed through reflexive distance from situations they have experienced with their illness (7–9). Experiential, clinical, and theoretical knowledge are not interchangeable; rather, they contribute distinct perspectives that, when brought into dialogue, can enrich one another. Involving people with experiential knowledge in co-production by complementary work has been shown to strengthen the co-produced health initiatives (4). These co-produced health initiatives reflect a better understanding of people’s experiences and need and, thus, have the potential to be more valuable and usable by the population (10–12). Moreover, participation as an experiential expert has been found to be beneficial for individuals themselves (13). When experiential experts participate as equal partners within redefined power-sharing practices, they experience development of skills and expertise, increased confidence and self-efficacy, and improved well-being (14, 15). These findings highlight that experiential knowledge is not only valuable for improving the relevance of health initiatives but also contributes to more empowering and transformative participation processes for those involved (4, 16).

In the context of RC the definition of co-production has evolved over time (17). In early studies on RC, authors presented co-production as doing things with people instead of doing things for them, which turned out to be a key feature of RC (18). For others, co-production in RC is a mechanism by which mental health service users experience different relationships with health care professionals (19). Some precisions about co-production are offered in a systematic review and qualitative study on RC by Crowther et al. (20). These authors defined co-production as individuals with experiential knowledge of mental health issues co-producing all aspects of RCs – from curriculum development to course delivery as well as operations and governance, alongside professionals (20). Co-production in RCs is also recognized for instilling a transformation of the way power is enacted and perceived, and the individuals involved experience a sense of equity of knowledge (21). This shift in power dynamics, the shared decision-making, and the recognition of the value of experiential knowledge in RC are also echoed in a recent systematic narrative review on co-production in Recovery Colleges (14).

In RC course development, the interaction between experiential knowledge (from lived experience), clinical knowledge (from clinical/field implication), and theoretical knowledge (from academia and research) gives rise to a form of collaborative work that is enacted through processes of mutual translation and negotiation. Within these processes, the trainers collaboratively articulate lived realities alongside theoretical and pedagogical approaches, contributing to the development of course content and activities that are both grounded in experience and pedagogically structured (22, 23). Vallarino and colleagues (2025) identified these practices as complementarity work. Through such interactions, complementarity work creates conditions for the emergence of new forms of knowledge that neither group could generate independently (16). However, achieving this complementarity requires specific conditions, including training and ongoing support, to enable meaningful participation and collaboration (24–26). While the Recovery College literature extensively documents the importance of co-production across governance, operations, and course delivery, several authors have highlighted the need for a more detailed understanding of how these processes unfold in practice (14, 20). In particular, they point to a lack of concrete and practical guidance to support the implementation of co-production, including structured processes, training, and ongoing support (20, 27, 28). Despite existing overviews, detailed empirical accounts of how co-production is operationalized in the development of Recovery College courses remain limited. Addressing this gap, the present article provides a concrete and in-depth example of a co-production process used to develop a course on eating attitudes and behaviors.

This article has a dual purpose. First, it aims to present a detailed account of the co-production process used to develop a Recovery College course on eating attitudes and behaviors through a three-phase participatory research approach. Second, it reports on the contributions of this process, examining the benefits of co-production for both the course itself and the experiential trainers involved.

## 2 Methods

### 2.1 Setting

This Recovery College (RC) course was developed at the first and only RC in Quebec (Canada), the Centre d’apprentissage santé et rétablissement (CASR). Established in 2019, the CASR emerged from a series of province-wide community consultations aimed at documenting the need for RC services among service users, community organizations, and healthcare network. These consultations identified strong interest in RC courses addressing a range of mental health and well-being topics, including eating attitudes and behaviors.

Experiential trainers—three individuals with lived experience of dysfunctional eating attitudes and behaviors—were recruited to attend the Train-the-Trainer program (26) prior to co-producing the course. As with all CASR courses, they were paired with a trainer with clinical and theoretical expertise (clinical trainer), in this case an occupational therapist specialized in eating attitudes and behaviors (who also attended Train-the-Trainer program). For the purposes of this study, and to document the co-production process from within, the clinical trainer also served as the principal researcher and first author of the present article (JT).

### 2.2 Positionality statement

The co-production of the RC course on eating attitudes and behaviors was conducted within the framework of a doctoral research project led by the first author (who is also a mental health occupational therapist). Throughout the study, the researcher occupied both insider and outsider positions, shifting between roles over the course of the research process (29, 30). As an insider, she supported the experiential trainers in the co-production of the course and acted as a Recovery College trainer with clinical and theoretical knowledge, co-facilitating the course alongside the experiential trainers. As an outsider, she was responsible for the design and conduct of the research, including data collection and analysis. This close engagement with the participatory process was explicitly acknowledged and addressed through the use of multiple data sources, the involvement of an independent interviewer during the initial implementation, and the validation of the analyses with a senior researcher (the thesis supervisor and second author) to ensure rigor and limit potential bias.

### 2.3 Research approach

The project adopted a participatory research approach. A participatory research approach involves a partnership between researchers and people who have interest in the research, but who are not researchers, referred to as stakeholders (31–33). The extent of stakeholder engagement is a key criterion for determining the participatory nature of a research project (34). In the context of this project, the co-production group—composed of the three experiential trainers and the clinical trainer (JT)—invited clinical experts in eating attitudes and behaviors, as well as CASR coordinators and senior staff members, to participate in a consultation group. This consultation group took part to a participatory co-validation process of the course content and learning activities, phase 2 of the study (described below).

Using a participatory research approach enabled the recognition and mobilization of experiential knowledge on an equal footing with clinical and theoretical knowledge (35). Moreover, this approach is consistent with core Recovery College principles as articulated in the literature (36–38).

### 2.4 Design and processes

The co-production of the RC course on eating attitudes and behaviors was guided by participatory design, a collaborative process that mobilizes the knowledge and expertise of users involved in the development of health and social innovations (39). Initially developed in the field of technology, participatory design employed a range of creative and interactive strategies (e.g., flash cards, colored sticky notes, cardboard mock-ups) to engage users at every stage of the co-production process (40). Nowadays, participatory design is recognized and applied in the development of many health and social initiatives (41). Participatory design is grounded in principles of equitable collaboration, in which users actively co-create and co-produce solutions alongside other stakeholders (40, 41). By valuing experiential knowledge and positioning users as equal partners, participatory design aligns closely with the co-production principles underpinning Recovery College.

The participatory design of this Recovery College course co-production was organized into three sequential phases: (1) a co-production phase based on a combination of collaborative and independent working sessions; (2) a co-validation phase involving a consultation group with mixed expertise; and (3) an initial experimentation phase with learners. Across all phases, the process was supported by the development and use of a series of tools, some dedicated to participatory design and others to research activities. **Table 1** provides an overview of each tool and the phase in which it was used. All tools are described in detail in Section 2.4.1.

**Table 1:** Overview of tools used according to each phases of participatory research.

Type of tool	Tool description	Phases of participatory research		
		Phase 1 co-production	Phase 2 co-validation	Phase 3 initial experimentation
Participatory design tools	Course co-production template	X	X	X
	Program theory model of the course (co-produced in the course development process)	X	X	X
Research tools	Research journal	X		
	Audit trail grid		X	
	Experimentation diary			X
	Individual interview guide			X

#### 2.4.1 Development and use of participatory design and research tools

To support the participatory process, the experiential trainers and the researcher used a set of co-participatory design tools to structure their collective work. In parallel, participatory research tools were employed to support data collection and analysis, with the aim of documenting the benefits of co-production strategies and tools for both the course being developed and the experiential trainers involved.

The participatory design tools consisted of a course co-production template and a program theory model of the course. The participatory research tools included a research journal, an audit trail grid, an experimentation diary, and an individual interview guide. Each tool is presented below.

##### 2.4.1.1 Participatory design tools

###### 2.4.1.1.1 Course co-production template

The course co-production template, a participatory design tool, was previously developed by the CASR team to support all trainers in the co-production of RC courses. Structured as a grid, the course co-production template allows trainers to specify each course session’s title and objectives, topics, evidence-based content, active learning activities, personal testimonies to share, and key take-home messages. As part of this study, all through co-production phase, the experiential trainers were responsible for completing the template. The experiential trainers and the researcher reviewed, discussed, and validated the template content.

###### 2.4.1.1.2 Program theory (logic) model of the RC course

The program theory model was developed in the course development by the clinical trainer (JT), building on the course co-production template completed by the experiential trainers. It was iteratively refined throughout the three phases of the project through feedback from the experiential trainers and the consultation group.

In line with program theory evaluation, a program theory model – or logic model- provides a graphical representation of the intervention (in that case the course on eating attitudes and behaviors) by illustrating the hypothesized relationships between the course objectives, activities, mechanisms of action, and expected outcomes (42, 43). More specifically, it maps how the course sessions, evidence-based content, active learning activities, and experiential testimonies are expected to contribute to proximal, intermediate, and distal benefits, ultimately supporting the adoption of functional eating attitudes and behaviors. Such a model serves both as a tool to support course development and as a guide for future evaluation by making explicit the intervention’s underlying assumptions and causal pathways (44, 45).

##### 2.4.1.2 Research tools

###### 2.4.1.2.1 Research journal

The researcher kept a research journal throughout the co-production phase. The journal documented decisions made during the process and the rationale behind them. Given the iterative nature of co-production, the journal systematically captured all changes and refinements to the course co-production template and the program theory model. It also served as a reflective strategy, enabling the researcher to document observations of the co-production process and to examine her insider/outsider roles and involvement. Entries from the journal were subsequently used as a source of data for analysis.

###### 2.4.1.2.2 Audit trail grid

The researcher completed an audit trail grid to document the co-validation phase during and after the consultation group meetings. The grid recorded all suggested improvements and modifications, noting both the rationale provided by consultation group members and how each suggestion was incorporated into the course co-production template, the program theory model, or both. The audit trail also served as a source of data for subsequent analysis.

###### 2.4.1.2.3 Experimentation diary

During the experimentation phase, the researcher completed an experimentation diary to document the course’s strengths, weaknesses, and areas requiring ongoing improvement. The diary was completed during and after each course session. Data from the diary were shared with the experiential trainers, allowing suggested improvements to be integrated into the course co-production template and program theory model throughout the experimentation. The diary thus served both continuous improvement and data analysis purposes.

###### 2.4.1.2.4 Interview guide

To document the experiential trainers’ experiences across all phases of the participatory research, individual semi-structured interviews were conducted during the experimentation phase. The researcher was responsible for the development of the interview guide. The participatory design tools (course template and program theory model) informed the development of the interview guide along with scientific literature on participatory research (31, 32). The interview guide was composed of open-ended questions and suggested prompts to facilitate discussion. Questions explored the experiential trainers’ perspectives on improvements made to the course, perceived benefits for learners and the course itself, the relevance of the content, and their experiences of co-producing and fine-tuning the course. Since the interview guide was not co-produced, it was piloted with members of the research team studying Quebec’s Recovery College that also includes one senior experiential trainer. The research team suggested minor modifications that were made accordingly. Given the researcher’s close involvement in the project, an independent interviewer conducted the interviews, each lasting approximately 60 minutes. All interviews were audio-recorded and transcribed verbatim for data analysis.

#### 2.4.2 Phase 1: co-production

The co-production process began with an initial meeting between the experiential trainers and the researcher (and clinical trainer, JT), aimed at collaboratively identifying the experiential trainers’ expectations and needs and establishing a working strategy for developing the RC course. Based on this discussion, a working strategy was adopted that combined collaborative working sessions (conveying the experiential trainers and the researcher) with alternating independent sessions involving only the experiential trainers as they expressed this need.

All collaborative sessions were audio-recorded to support the completion of the research journal. During the independent sessions, the experiential trainers completed the course co-production template, outlining potential topics, content, and activities for each session, while the researcher focused on maintaining the research journal and drafting the program theory model of the RC course.

#### 2.4.3 Phase 2: co-validation

Following the completion of phase 1, an additional co-validation phase was conducted. In the context of this research co-validation refers to the collective review and acceptance of the course on eating attitudes and behaviors as legitimate and coherent with the RC principles. This step is not typically included in the co-production of other CASR courses but was implemented due to the sensitive nature of eating attitudes and behaviors. Current recommendations in health education emphasize promoting protective factors that support functional eating attitudes and behaviors rather than focusing on disordered eating (46, 47).

In line with these recommendations, a consultation group with mixed expertise was established to co-validate the course and its program theory model. The group comprised the experiential trainers, the researcher, two members of a research team specializing in eating attitudes and behaviors, and two members of a research team specializing in RC. This multi-expertise representation ensured the integration of experiential, clinical, and theoretical knowledge across both areas of focus (eating attitudes and behaviors and RC).

The consultation group participated in a series of three online meetings, interspersed with collaborative working sessions involving only the experiential trainers and the researcher. During the online meetings, the group reviewed the course co-production template and program theory model, suggesting improvements and modifications along with their rationale. The experiential trainers and researcher responded to these suggestions. Differences of perspective were brought up in the consultation group and discuss collectively to reach a consensus guiding agreed-upon changes. This consensus sought to changes that both supported evidence-based practices in EAB and RC principles.

The audit trail grid was used to systematically document the co-validation process. Following each online meeting, the experiential trainers and researcher integrated the agreed-upon modifications into the course template and program theory model during collaborative working sessions. Updated versions were then shared with the consultation group at the start of the subsequent meeting. All online meetings were audio-recorded to support completion of the audit trail grid. **Figure 1** presents the co-validation phase and the use of participatory design and research tools.

**Figure 1:**
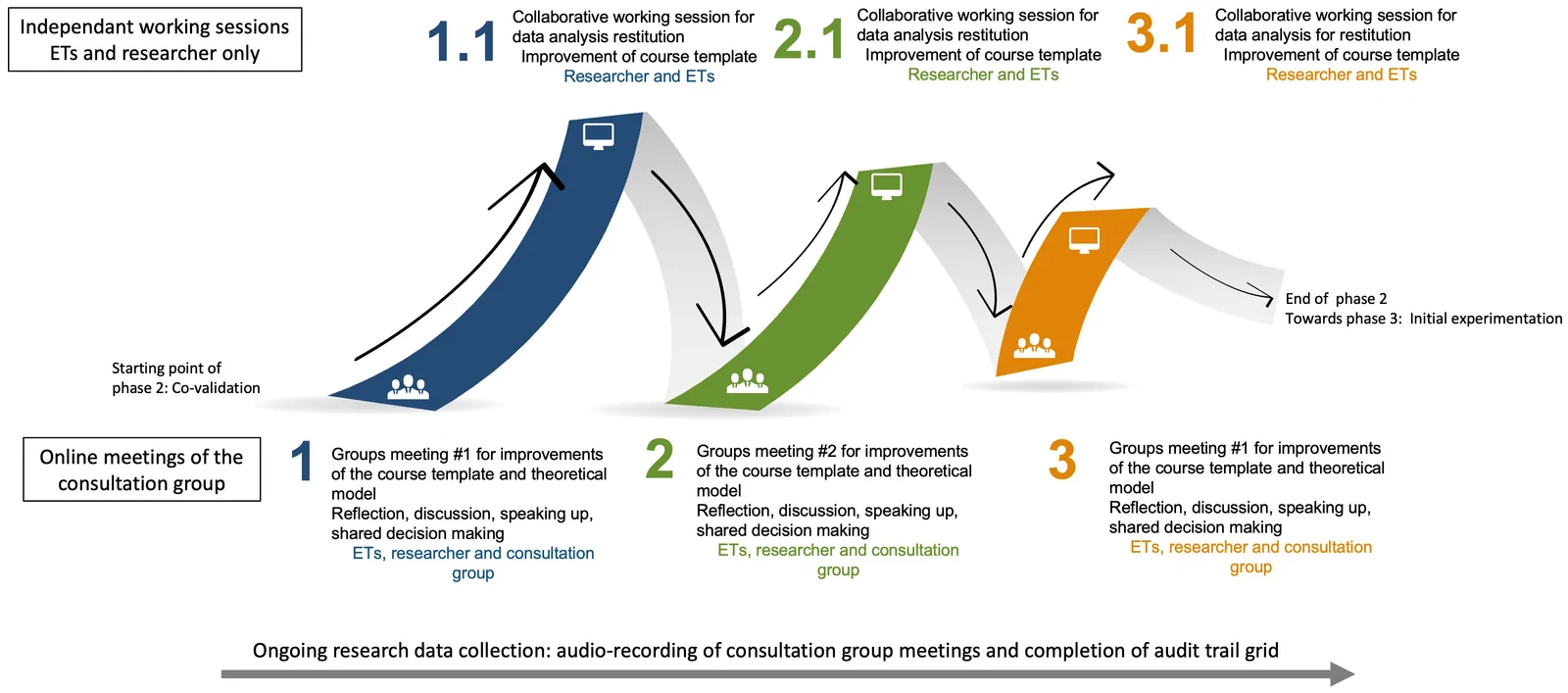
Presentation of phase 2: co-validation.

#### 2.4.4 Phase 3: initial experimentation

Following the co-validation phase and subsequent revisions to the course template and program theory model, the course was implemented at CASR for initial experimentation with learner groups. The course comprised three two-hour sessions per week, for a total of six hours. Over one year, four groups participated across four semesters.

Each semester, the course was co-facilitated by one experiential trainer and the researcher (with clinical and theoretical knowledge), forming a facilitator dyad. In practice, two experiential trainers co-facilitated one course each with the researcher, while one experiential trainer co-facilitated two. After the first semester, the three experiential trainers took part to an individual interview to document their experience of taking part to the three phases (co-production, co-validation, initial experimentation).

Throughout each semester of the initial experimentation, the researcher completed an experimentation diary to document the course’s strengths, weaknesses, and areas requiring improvement. The experiential trainers incorporated refinements throughout the experimentation, which the researcher also integrated into the program theory model. **Figure 2** presents the unfolding of phase 3 over four semesters. Each semester, learners were invited to take part to individual interview to document their experience of the course and learning outcomes achieved. These findings will be presented in a subsequent article.

**Figure 2:**
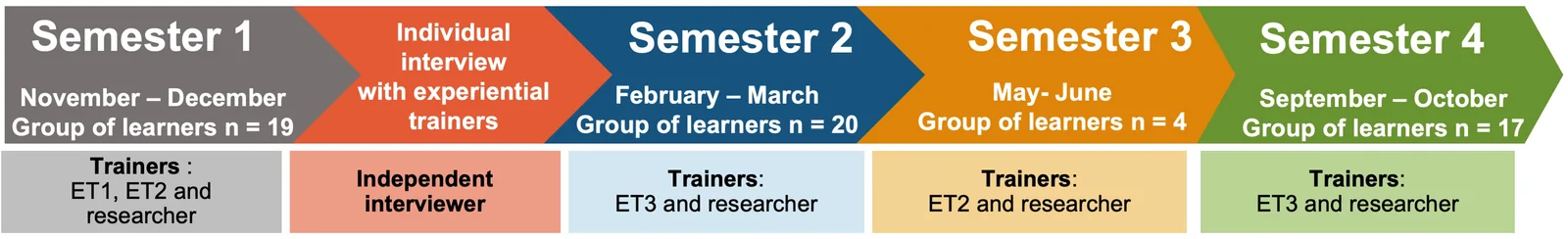
Presentation of phase:3 Initial experimentation.

### 2.5 Data analysis

A substantial and diverse corpus of qualitative data was generated for this study. The data included: collective sessions audio and the research journal kept during phase 1; audio recordings of the consultation group meetings and the audit trail grid from phase 2; transcriptions of individual interviews with the experiential trainers, and the experimentation diary completed during phase 3.

The data were analyzed using an analytical inquiry strategy (48), a method particularly suited for large and heterogeneous qualitative datasets. Unlike traditional coding approaches, analytical inquiry involves systematically “questioning” the material. Through meticulous examination, a series of queries are applied iteratively, with deeper questions emerging as preliminary conclusions are drawn, ultimately generating comprehensive insights. To ensure rigor, analysis was conducted in ongoing consultation with a senior researcher (CB), where divergent interpretations were openly discussed and common understanding was sought. The findings from these queries were synthesized into conclusions, which were then organized into thematic categories.

The analytical process followed a sequential approach aligned with the three phases of the project. First, conclusions were drawn from the collaborative sessions’ audio and the research journal documenting the co-production phase in order to highlight the needs, processes and worked accomplished between the experiential trainers and the researcher. Second, data from the co-validation phase—audio recordings of consultation group meetings and the audit trail grid—were subjected to queries designed to evaluate how course improvements strengthened content and activities, reinforced theoretical soundness, and ensured coherence with Recovery College principles. These conclusions were then organized into thematic categories. Third, data from the initial experimentation phase—the experimentation diary and interview transcripts—were analyzed to examine improvements made to the course and the experiential trainers experiences as equal partners in the co-production and co-validation processes. **Figure 3** presents the analytical inquiry strategy flowchart.

**Figure 3:**
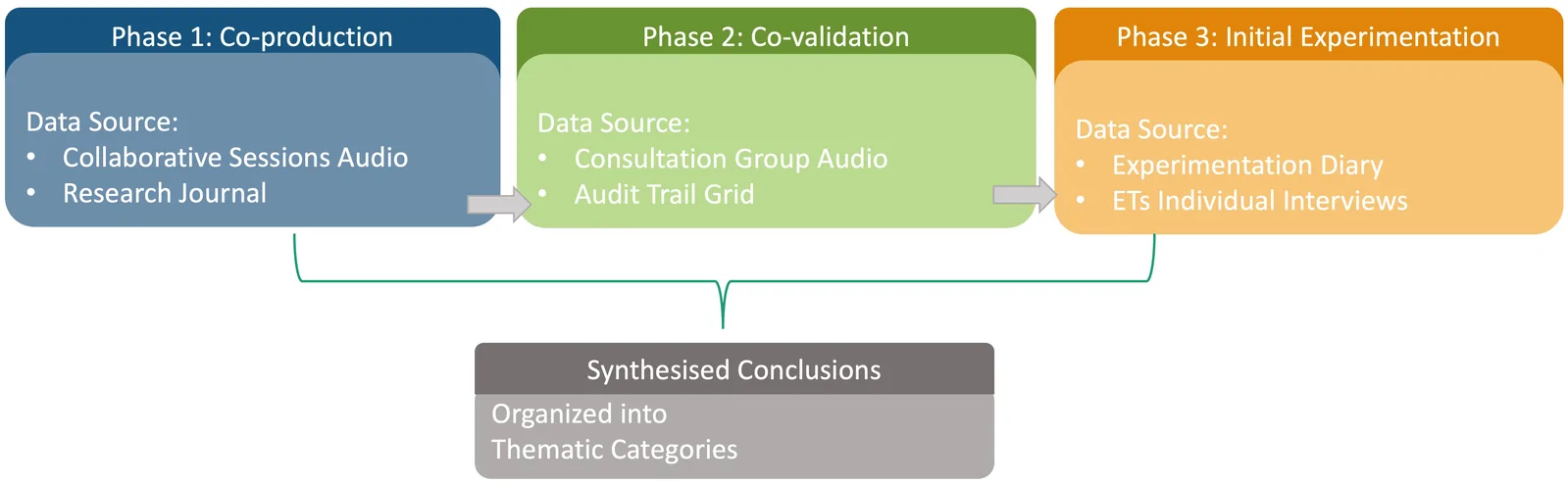
Analytical inquiry strategy – flowchart.

### 2.6 Ethics considerations

The project complies with the Declaration of Helsinki (49) and obtained approval of the Université du Québec à Trois-Rivières research and ethics committee (certification number CER-21-281-07.06).

## 3 Results

Before presenting the results, it is important to restate the study’s objectives which are to (1) present a three-phases participatory co-production process used to develop a Recovery College course on eating attitudes and behaviors and (2) report on the results of this process, examining the benefits of co-production for both the course itself and for the trainers involved. The results are presented according to these three phases, highlighting contributions to course improvements, theoretical soundness, and experiential trainers’ engagement.

### 3.1 Phase 1: co-production

The co-production phase involved five collaborative working sessions (with the experiential trainers and researcher) and five independent sessions (experiential trainers only), totaling approximately 10 hours each. **Figure 4** presents the sequence of this working strategy

**Figure 4:**
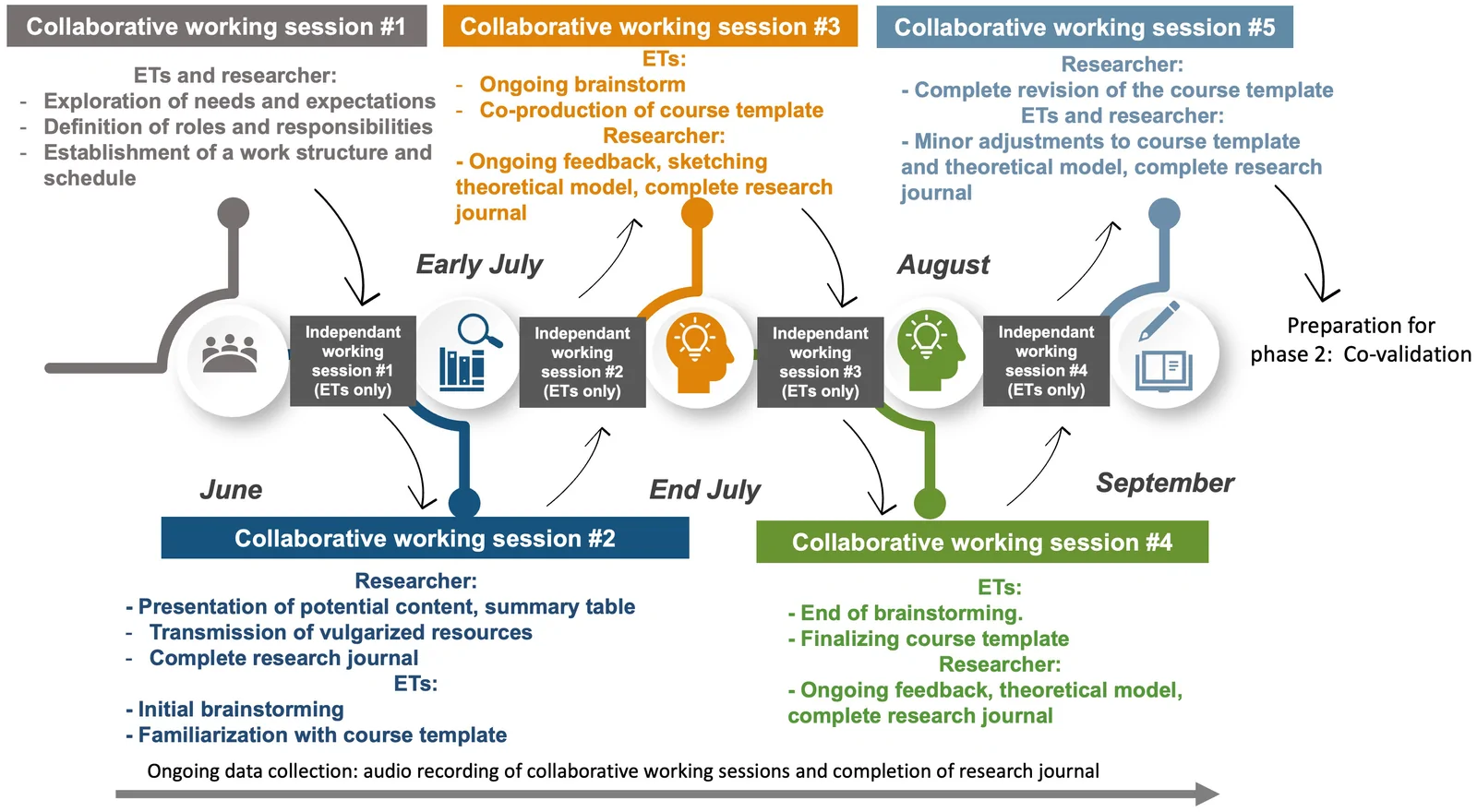
Results of phase1: co-production.

This working strategy was adopted in response to the experiential trainers’ expressed need for a balance between support and autonomy throughout the co-production process. The experiential trainers also indicated a desire for accessible, evidence-based content on eating attitudes and behaviors. In response, the researcher conducted a targeted review of the scientific literature during the co-production phase and shared relevant materials with the experiential trainers during the collaborative sessions, including summaries, PowerPoint slides, and selected articles.

*“Accompanying the experiential trainers during the co-production phase requires intensive and sustained involvement. It calls for a facilitative leadership approach that involves tailoring the support to the needs of the experiential trainers. This takes the form of negotiating a working strategy that recognizes their expertise and autonomy, while ensuring access to high-quality information presented in an understandable and accessible manner.”*

Research journal entry – independent working session #1

During the co-production phase, the experiential trainers expressed a need for guidance and validation in selecting session topics, content, and activities. In response, a collaborative working session was specifically dedicated to addressing this request. This support enabled the experiential trainers to complete an initial version of the course template, which included the course title, four objectives, and four topics. Each topic integrated evidence-based content, active learning activities, and personal testimonies drawn from the experiential trainers’ lived experience. Time for open discussion with learners was also built into the course to allow participants to share their experiences and reflections.

*“An important responsibility is to maintain ongoing reflections with the experiential trainers regarding the selection of course topics, content, and activities. This required, at times, reorienting certain choices to ensure alignment with the scientific literature on eating attitudes and behaviors.”*

Research journal entry – independent working session #3

Based on this initial version of the course template, the researcher created an initial version of the program theory model of the course. The initial version of the program theory model hypothesized links between the course’s objectives and its components (topics, evidence-based content presented, activities, and testimonies) to achieve the desired outcomes. **Figure 5** presents this initial version of the program theory model which underwent improvements in the co-validation phase process.

**Figure 5:**
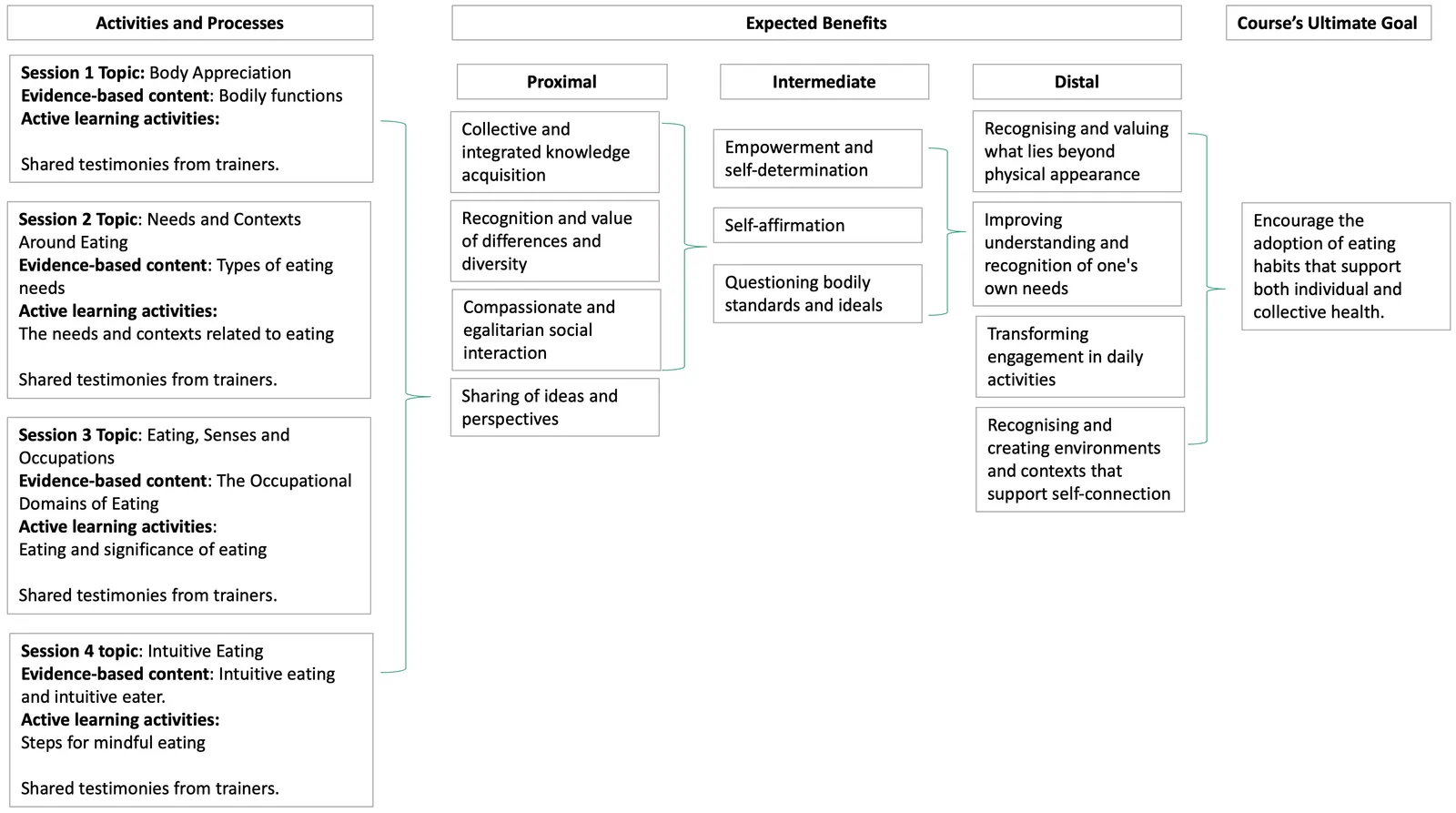
Initial program theory model of the course – prior to phase 2: Co-validation.

### 3.2 Phase 2: co-validation

The co-validation process involved three two-hour online meetings bringing together the experiential trainers, the researcher, and the consultation group. In addition, the experiential trainers and the researcher met between meetings to revise the course materials based on the consultation group’s recommendations. In total, 14 hours of work were devoted to the co-validation process.

The audit trail grid documenting the suggested improvements and modifications, along with the audio recordings of the meetings, constituted the data corpus for this phase. This material was first examined through an initial analytical query: *“What is the impact of the suggested improvements on the course?”* The results of the subsequent deepening queries are presented in **Table 2**.

**Table 2:** Results of qualitative analytical inquiry of the co-validation process.

Initial query: What is the impact of the suggested modifications on the course?		
Deepening queries	Concluding results	Category
How do these changes influence the course’s coherence?	• Improved alignment between objectives, session topics, evidence-based content, active learning activities, and testimonials. • Enhanced use and consistency of evidence-based content in course materials. • Updated program theory model and course template with coherent content. • Strengthened overall coherence between course components and the intervention rationale.	Enhanced content coherence
In what ways do the proposed improvements enhance the course’s precision?	• Increased precision and consistency of terminology in all course content. • Standardized terminology usage by replacing synonymous or redundant terms	Enhanced precision of terminology across all supporting materials
How do the suggested changes improve the course’s clarity?	• Clarified complex terms through refined explanations and definitions. • Added concrete, relatable examples to facilitate comprehension.	Improved clarity for ETs and learners
What prompted the proposed adjustments to the integration of experiential knowledge?	• Explicitly encouraging sharing of experiential knowledge by learners. • Ensuring adherence to the principle of equality between experiential and scientific knowledge. • Removing non-essential evidence-based content to balance perspectives. • Reallocating time to active, discussion-based learning. • Expanding opportunities for learners to share experiential insights.	Improved compliance with the principle of equality of knowledge

#### 3.2.1 Enhanced content coherence

The analysis of the consultation group meetings underscored the need to strengthen the internal coherence of both the program theory model and the course template. Adjustments were recommended to ensure closer alignment between the learning objectives, session topics, evidence-based content, activities, and testimonials, and the scientific literature underpinning the intervention. In particular, the consultation group emphasized the importance of using consistent terminology drawn from the evidence base across all course materials. They also stressed that any adjustments made to the program theory model should be systematically reflected in the course template, and vice versa, to maintain coherence between these two foundational documents.

*“Use the same content across all course-related documents. Particular attention should be given to ensuring consistency between the course template and the program theory model. This enhances the overall coherence of the course and demonstrates the rigor of its development”.*

Audit trail grid entry – group meeting #1

#### 3.2.2 Enhanced precision of terminology across all supporting materials

The analysis highlighted the need to strengthen the precision of the course content through consistent terminology. The consultation group noted variations in wording that could create confusion in theoretical components and active learning activities. To address this, they recommended selecting a single term for each concept and applying it consistently across all course materials. They also emphasized that these adjustments should be systematically incorporated into both the course template and the program theory model to ensure clarity and coherence for learners.

“*Use the same vocabulary throughout all course-related documents. Consistency in terminology increases precision and should be maintained for the course objectives, theoretical content, and active learning activities. This consistency should be reflected both in the course template and in the program theory model of the course.”*

Audit trail grid entry – group meeting #2

#### 3.2.3 Improved clarity for trainers and learners

The analysis highlighted the need to enhance the overall clarity of the course for both learners and trainers. The consultation group noted that some learners might encounter certain terms for the first time, emphasizing the importance of providing simplified explanations or lay definitions where needed. Particular attention was given to the course title and brief description, which were found to be overly complex or not sufficiently concrete. The group recommended reformulating the title and simplifying the description using plain, accessible language. They also stressed the importance of defining key terms in evidence-based content and active learning activities and suggested including concrete, relatable examples to illustrate complex concepts, thereby improving both clarity and relevance for all learners.

*“Some terms in the title are complex or difficult to understand. While they may have been chosen to make the title more striking, they are not clear to me and may be confusing for potential learners”*

Member of consultation group – audio recording group meeting #3

#### 3.2.4 Improved compliance with the principle of equality of knowledge

The analysis emphasized the importance of ensuring that experiential, clinical, and theoretical knowledge were treated as equally valuable throughout the course. The consultation group recommended systematically integrating insights from the experiential trainers alongside evidence-based content in both the course template and program theory model. This approach reinforced the participatory and inclusive principles of the Recovery College model and ensured that all forms of knowledge were recognized and reflected in the course design.

*“There is a substantial amount of theoretical, evidence-based content presented. Reflections should focus on reallocating time to reduce theoretical content in favor of active learning activities, open discussion, and experience sharing, which are more closely aligned with Recovery College principles.”*

Audit trail grid entry – group meeting #3

Taken together, these recommended adjustments strengthened the course’s components by enhancing terminological precision and ensuring greater alignment between theoretical content and pedagogical activities. By reinforcing adherence to the principle of equality of knowledge, the adjustments more effectively integrated experiential and theoretical perspectives within a cohesive course framework. Collectively, these improvements informed the revision of the initial program theory model, culminating in an updated, more conceptually robust version presented in **Figure 6**.

**Figure 6:**
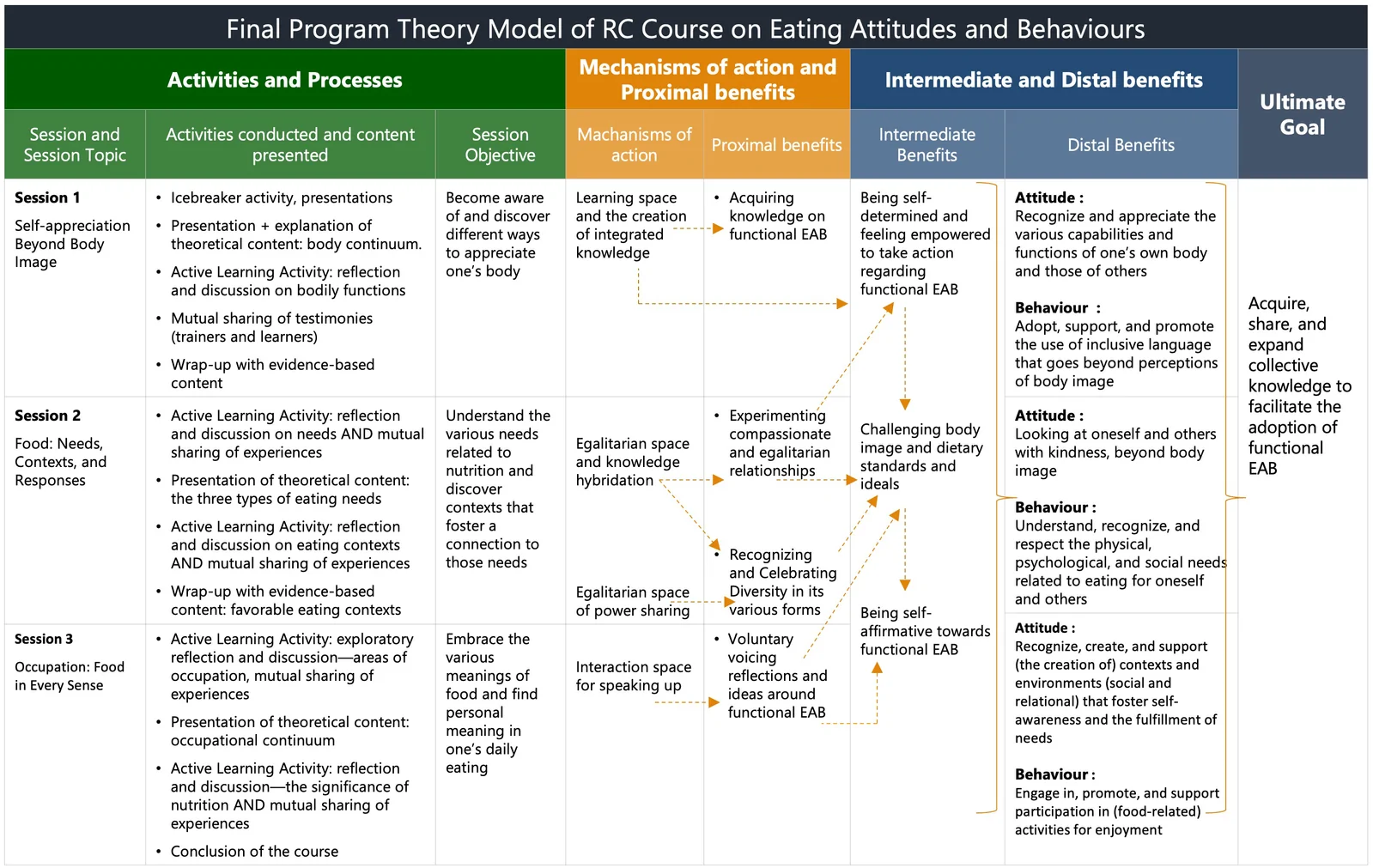
Final program theory model of the course – edited after phase 2: co-validation.

### 3.3 Phase 3: initial experimentation

The initial experimentation of the course took place over nearly one year, across four semesters. The three experiential trainers and the researcher alternated as co-facilitators for the course, which was attended by four groups of learners, totaling 60 participants. The data corpus for this phase included the experimentation diary and the transcripts of the individual interviews with the three experiential trainers. This material was analyzed using the analytical inquiry strategy, beginning with the initial query: *“How did implementing the suggested improvements prove beneficial?”* The results of the subsequent deepening queries are presented in **Table 3**.

**Table 3:** Results of qualitative analytical inquiry of the initial experimentation.

Initial query: How did implementing the suggested improvements turned out to be beneficial?		
Deepening queries	Concluding results	Category
How has the improved content benefited the course?	• Providing details and nuances in the evidence-based content of the course was perceived to enrich the course itself and the learners ‘experience	Added details and nuances to the course
What was the rationale for detailed and nuanced content?	• Enriching the content presented and the learning activities keeps the alignment with the evidence-based information supporting the course • These modifications gave confidence to the ETs that their course will increase the understanding and uptake for the learners • The ETs felt these changes made the course more inclusive	Enriched content for increased understanding and inclusivity
What is the takeaway of being involved in all these collaborative processes	• Being involved and recognized as experiential trainers in all aspects of co-production and co-validation increase knowledge and skills in course facilitation • This involvement offers validation and recognition of the experiential knowledge of the ETs	Increased knowledge and skills: the value of recognizing experiential knowledge.

#### 3.3.1 Details and nuances

From the experiential trainers’ perspective, the course improvements primarily involved adding details and nuances to the theoretical content. Clarifying the meaning of key terms, such as eating attitudes and behaviors, helped learners better understand the overall course structure. In line with the consultation group’s recommendations for clearer explanations, the experiential trainers express that these additions stimulated reflection and fostered richer discussions among learners. They add that the adjustments also prompted the trainers to diversify active learning activities, reaching a broader range of learners. Overall, these refinements enhanced the course by improving comprehension, inclusivity, and learners’ engagement.

*“Following the consultation group meetings, there was certain content which I realized I wasn’t really understanding the goal and objective behind its presentation or why we were talking about it. So, we ended up adding explanations. I really think that helped learners understand where we were heading in that session. Then we did an activity on that content, and I think it made things clearer.”* — Experiential trainer 3

#### 3.3.2 Enriched content for increased understanding and inclusivity

Efforts to enrich the theoretical content also made the course more comprehensive and inclusive. The experiential trainers reported that these additions promoted a deeper understanding among learners by clarifying expectations and improving comprehension, notably through the integration of both textual and visual materials. Expanding the information presented allowed the experiential trainers to establish stronger connections between the theoretical content they shared and the active learning activities.

*“You know, we had talked about it. For me, it was important that we present the graphic—the model of eating attitudes and behaviors. It was essential to synthesize and simplify it for the learners so they could understand the orientation of the course and why we made these content choices.”* — Experiential trainer 1

Adding nuance to the course content enabled the experiential trainers to adopt a more inclusive pedagogical approach. Inclusivity, for the trainers, involved acknowledging and addressing the diverse characteristics and needs of learners. Accordingly, they refined the content and examples shared and actively encouraged learners to express their own varied perspectives.

*“I didn’t really recognize myself in some of the theoretical content and examples shared. So, we reflected on this, and we discussed how important it is to add nuance—to also share examples of strategies that didn’t work for us and what we did to find things that do. That makes it more diverse, and learners are more likely to see themselves and feel included.”* — Experiential trainer 3

#### 3.3.3 Increased knowledge and skills: the value of recognizing experiential knowledge

Reflecting on the three phases (co-production, co-validation and experimentation) and their personal gains, the experiential trainers described significant learning and transformation that supported their own recovery process. They characterized the experience as deeply enriching, noting that they learned as much as the participants. The trainers reported gaining knowledge about evidence-based information on eating attitudes and behaviors, pedagogical practices, and insights into themselves. They also felt validated throughout the process and recognized the meaningful contribution of sharing their lived experiences. A sense of usefulness and acknowledgment of their experiential knowledge emerged as a key outcome of the co-production and experimentation process.

*“I think it taught me a lot. I really experienced personal learning. It helped me align better with my own recovery because I still tend to have concerns about my physical appearance. That’s something I’d like to gradually change.”* — Experiential trainer 2

*“It really opened my eyes. It helped me become more aware and make changes in my daily life. It also helped me let go of guilt—and that’s really thanks to the course.”* — Experiential trainer 3

*“I felt that I could bring something to the group. [My contribution wasn’t only through presenting theory.] I could contribute [to learners] through my experience, my lived knowledge, and my sharing … [Also], I felt that while I was learning, I was being validated by talking about it. It’s helpful—it really feels good.”* — Experiential trainer 1

Overall, the experiential trainers’ reflections support the added value of a structured participatory design process for Recovery College course co-production and reinforce the recommendations and modifications made by the consultation group. Their testimonies illustrate how the adjustments—particularly those enhancing content, inclusivity, and recognition of experiential knowledge—translated into tangible improvements in the course. By refining both the theoretical and pedagogical components, the trainers not only strengthened learner engagement but also experienced meaningful personal growth and validation of their lived expertise.

## 4 Discussion

This detailed account of a three-phase participatory design process for the co-production of a Recovery College course on eating attitudes and behaviors highlights its methodological and empirical contributions and provides a practical example of participatory course development.

### 4.1 Key practices for operationalizing complementarity work in co-production

As noted by some scholars in the field of Recovery College, it is sometimes difficult to know how to bring together different forms of knowledge and expertise, as well as where to begin the co-construction process (20). To support the co-production phase and translate complementarity work into concrete process, this study highlights specific practices as key: open discussion for work structure and role definition, and the use of participatory tools.

#### 4.1.1 Discussion for work structure and role definition

Open discussions about the experiential trainers’ needs, expectations, and preferred forms of support helped to shape the direction of the co-production process. These discussions informed the development of a work structure which created a protected space dedicated to the experiential trainers. The establishment of a safe space between the trainers have been identified as a key element supporting co-production practices (16). Creating a work structure that protected independent work sessions conveying only the experiential trainers supported their autonomy to develop course content while being able to mobilize contributions from the researcher as needed. Having open discussions and planning a work structure constitute concrete practices of co-production process that help bringing together different forms of knowledge and expertise. Such an approach also reflects core Recovery College principles of co-production, particularly in relation to power-sharing and the recognition of experiential expertise, by enabling both independence and collaboration within the process (18, 24). These discussions also helped clarify roles within the co-production process. Clearly defining roles revealed to be an important aspect of co-production, as it supported the articulation of distinct yet interdependent contributions. Previous work highlights that careful consideration to role definition is an important component of co-production in Recovery College (22, 50). Delineating roles and responsibilities can help mitigate potential ambiguities related to power and legitimacy within the co-production process (25). In the context of this Recovery College course, clearly defined roles contributed to mobilizing experiential, clinical and theoretical knowledge in complementarity work.

#### 4.1.2 Participatory design tools to support co-production

The use of participatory design tools also played a key role in supporting the co-production phase. The course template and the program theory model facilitated the appropriation of the process for both the experiential trainers and the researcher by providing a shared understanding to guide course development. In their recent scoping review, Dalgarno and colleagues (2025) noted that more training and guidance were required to co-production processes in RC. Our results illustrate that these tools supported co-production process by bridging experiential and theoretical perspectives, making them more articulable within the co-production process. Recent international research between Quebec and Italy identified that using participatory tools, supports Recovery College trainers in deploying co-production practices aligned with the core principles of Recovery College (16). In the current project, using participatory design tools is a concrete practice through which co-production was operationalized, as it enabled the experiential trainers to contribute in ways that value and recognize their contribution and unique knowledge. In our perspective, this constitutes a strength of this research. The use of such tools was part of a training program for trainers but also required careful facilitation to ensure that they do not constrain the expression of experiential knowledge or impose overly rigid frameworks on the co-production process. As previously advocated by Briand et al. (51), research efforts around co-production practices and tools in Recovery College should be maintained.

### 4.2 Strengthening capacities through co-production and co-validation

The integration of co-production and co-validation processes contributed to strengthening capacities at both the intervention and trainers’ levels. By supporting the refinement of course content alongside the recognition and development of experiential expertise, these processes enabled a dual outcome of enhanced course quality and trainers’ recognition and empowerment.

#### 4.2.1 Quality criterion for course development

Beyond supporting the co-production process itself, the inclusion of a co-validation phase with a consultation group appears to have played a critical role in strengthening the quality and rigor of the course being developed. While co-production enabled the integration of experiential and theoretical knowledge, the co-validation process contributed to refining this integration by enhancing the internal coherence of the course components, improving the precision of terminology, and increasing overall clarity for both trainers and learners. These findings suggest that co-validation can support a more systematic alignment between learning objectives, theoretical content, and active learning activities, thereby reinforcing the consistency and accessibility of the course, which are key elements in the development of effective educational initiatives (28, 52). In addition, the adjustments made through co-validation contributed to a better alignment with Recovery College principles, particularly regarding the recognition of the equality of knowledge, by ensuring a more balanced integration of experiential and evidence-based content as suggested in previous work (14, 20, 53).

#### 4.2.2 Driver for experiential knowledge recognition

In line with participatory design approaches, particular attention was paid throughout the three phases to ensuring the active involvement of experiential trainers and supporting their understanding and appropriation of the process (35, 42). They reported several benefits of their involvement in this Recovery College course development process. They perceived their involvement as enriching and contributing to a sense of recognition and validation of their experiential knowledge. This engagement also supported learning, both in relation to functional eating behaviors and to evaluation and co-production processes, while fostering a sense of equality within the triad of experiential trainers. The trainers highlighted their ability to contribute meaningfully at both personal and professional levels, reinforcing their confidence and perceived capacity to support others. These findings echo previous research on Recovery Colleges, which highlights the positive impacts of participation for individuals with lived experience, including increased self-efficacy, skill development, and empowerment (27, 54). Taken together, these results suggest that combining co-production with co-validation processes not only strengthens the intervention itself but also creates conditions that support the meaningful engagement and development of experiential trainers. This finding aligns with broader calls for more structured and iterative approaches to co-production in health research and service development (5, 6).

### 4.3 Limits and strengths

This study presents some limitations that should be considered when interpreting its findings. First, the co-production and co-validation processes described are time- and resource-intensive, requiring sustained engagement from experiential trainers, researchers, and consultation group members, as well as organizational support. In practice, Recovery Colleges and other organizations may face constraints in transferring such approaches, highlighting the need to explore how co-production can be adapted while maintaining meaningful participation. Second, despite efforts to foster shared decision-making, questions regarding decision-making authority remained a grey area. While the consultation group provided guidance and validation, who ultimately held decision-making power was not always explicit, reflecting broader challenges related to power-sharing and role negotiation in co-production. Third, although beneficial, the co-validation phase required careful facilitation to ensure that feedback did not undermine experiential knowledge but rather supported and enhanced it, underscoring the delicate balance between validation and recognition of experiential expertise. Fourth, involving a lived experience expert in the analysis process or seeking a formal confirmation of authenticity of the results from the experiential trainers would have strengthen the analysis. Finally, the researcher’s close involvement throughout the process also warrants consideration. Acting in a dual role as facilitator and researcher supported trust-building and responsiveness but may have influenced collaboration dynamics and data interpretation. While efforts were made to ensure reflexivity and transparency, this positionality remains an important consideration.

This study also presents several strengths that contribute to its relevance and rigor. First, it provides a rare and detailed empirical account of a co-production process in a Recovery College context, including the integration of a co-validation phase. While co-production is widely advocated in the literature, detailed descriptions of how such processes are operationalized in practice remain limited. By documenting a structured three-phase participatory approach, this study offers concrete insights that can inform the development of similar initiatives in other settings. Second the study demonstrates the added value of combining co-production with co-validation processes. While co-production supported the co-construction of course content, the co-validation phase enabled its critical refinement, enhancing coherence, clarity, and alignment with evidence-based knowledge. This combined approach represents a novel contribution to the field and offers a promising avenue for strengthening participatory practices in health education and service development. A third strength of this study lies in the use of a range of participatory design tools that supported both the co-production process and the research process. Tools such as the course template and the program theory model facilitated collaboration by providing a shared framework through which experiential and theoretical knowledge could be articulated and integrated. At the same time, research-oriented tools, including audit trails and documentation of group discussions, contributed to the transparency, traceability, and rigor of the study. The combined use of these tools supported both the operationalization of co-production and the systematic analysis of the process, strengthening the credibility of the findings. This dual function highlights the value of integrating practical and methodological tools in participatory research to support both meaningful collaboration and robust knowledge production.

## 5 Conclusion

This study highlights that co-producing a health initiative, whether within a Recovery College or other contexts, requires the development of strong and intentional partnerships supported by concrete strategies and tools. The findings suggest that combining co-production with co-validation processes can strengthen both the quality of the initiative and the capacities of those involved, while fostering collaboration by bringing together differing perspective and knowledge. In this regard, the development and use of tools to support co-production represent a promising avenue for Recovery Colleges and similar initiatives, as they help structure collaboration, promote learning, and support more equitable participation among stakeholders. Ultimately, this study underscores that meaningful co-production is not solely based on participation, but on the ability to create conditions—through practices, structures, and tools—that enable partners to work together on an equal footing toward shared objectives.

## Data Availability

The raw data supporting the conclusions of this article will be made available by the authors, without undue reservation.
